# Evaluation of Operator Variability and Validation of an AI-Assisted α-Angle Measurement System for DDH Using a Phantom Model

**DOI:** 10.3390/bioengineering12091004

**Published:** 2025-09-22

**Authors:** Yusuke Ohashi, Tomohiro Shimizu, Hidenori Koyano, Yumejiro Nakamura, Daisuke Takahashi, Katsuhisa Yamada, Norimasa Iwasaki

**Affiliations:** 1 Department of Orthopaedic Surgery, Faculty of Medicine and Graduate School of Medicine, Hokkaido University, Sapporo 060-8638, Japan; yuk1274go@gmail.com (Y.O.); nakamurayjiro33@gmail.com (Y.N.); rainbow-quest@pop02.odn.ne.jp (D.T.); yka2q@pop.med.hokudai.ac.jp (K.Y.); niwasaki@med.hokudai.ac.jp (N.I.); 2 Department of Medical Physics, Graduate School of Medicine, Hokkaido University, Sapporo 060-8638, Japan; koyano@med.hokudai.ac.jp

**Keywords:** Developmental Dysplasia of the Hip (DDH), ultrasound imaging, Artificial Intelligence (AI), α-angle measurement, operator variability

## Abstract

Ultrasound examination using the Graf method is widely applied for early detection of developmental dysplasia of the hip (DDH), but intra- and inter-operator variability remains a limitation. This study aimed to quantify operator variability in hip ultrasound assessments and to validate an AI-assisted system for automated α-angle measurement to improve reproducibility. Thirty participants of different experience levels, including trained clinicians, residents, and medical students, each performed six ultrasound scans on a standardized infant hip phantom. Examination time, iliac margin inclination, and α-angle measurements were analyzed to assess intra- and inter-operator variability. In parallel, an AI-based system was developed to automatically detect anatomical landmarks and calculate α-angles from static images and dynamic video sequences. Validation was conducted using the phantom model with a known α-angle of 70°. Clinicians achieved shorter examination times and higher reproducibility than residents and students, with manual measurements systematically underestimating the reference α-angle. Static AI produced closer estimates with greater variability, whereas dynamic AI achieved the highest accuracy (mean 69.2°) and consistency with narrower limits of agreement than manual measurements. These findings confirm substantial operator variability and demonstrate that AI-assisted dynamic ultrasound analysis can improve reproducibility and reliability in routine DDH screening.

## 1. Introduction

Although ultrasound examination using the Graf method is widely employed for the early detection of developmental dysplasia of the hip (DDH), concerns regarding the reliability of its measurements remain [1,2]. Both intra-operator variability—where repeated measurements by the same examiner yield inconsistent results—and inter-operator variability—differences between examiners—have been recognized as significant limitations. Contributing factors include probe angulation, applied pressure, transducer positioning, and subjective interpretation of anatomical landmarks [3,4,5]. Even experienced examiners are susceptible to such variability, and prior studies have demonstrated that key diagnostic parameters, particularly the α- and β-angles, are highly sensitive to subtle deviations in technique [4,6]. Despite efforts to reduce these discrepancies through standardized training, a moderate degree of inconsistency persists, underscoring the inherent difficulty in achieving fully reliable and reproducible ultrasound-based assessments of the infant hip [3,7].

In response to these challenges, recent research has turned to artificial intelligence (AI) as a means to improve diagnostic consistency and reduce operator dependence in DDH ultrasound assessments [8]. Deep learning–based models have shown promising performance in automating critical components of the Graf classification, including landmark detection, standard plane identification, and α-angle measurement, with diagnostic accuracy comparable to that of expert clinicians [9,10,11]. These AI-assisted systems offer the potential to standardize assessments, improve reproducibility, and expand access to high-quality screening. Previous AI-assisted approaches for DDH ultrasound have primarily relied on static image analysis, often using manually selected frames to detect anatomical landmarks or estimate α-angles. While these studies demonstrated the feasibility of applying deep learning to DDH diagnosis, they did not capture operator-dependent variability or provide real-time diagnostic support. Our study builds on this body of work by introducing a dynamic, video-based AI system capable of frame-by-frame α-angle measurement during continuous scanning and validating its accuracy against a phantom with a known reference angle. This provides both real-time applicability and objective benchmarking that have been lacking in previous reports. However, before the benefits of such technologies can be accurately evaluated, it is essential to establish a quantitative understanding of the variability that exists among human examiners with different levels of training and experience.

To that end, we designed a two-part study to explore these issues. First, we conducted a phantom-based experiment (Study 1) to assess diagnostic accuracy and examination time among medical students, residents, and experienced physicians, thereby quantifying intra- and inter-operator variability under controlled conditions. Second (Study 2), we developed and validated an AI-based system capable of automatically selecting diagnostic-quality frames and calculating α-angles from both static ultrasound images and video sequences.

Previous studies have mainly addressed operator variability or AI-assisted systems in isolation, without integrating both perspectives within a unified framework. This has left two key gaps: (1) a limited quantitative understanding of how examiner experience affects measurement accuracy and reproducibility, and (2) insufficient validation of AI-assisted methods against objective reference standards under controlled conditions. Our two-part design was chosen specifically to address these gaps: Study 1 established a benchmark of human performance, and Study 2 directly evaluated an AI-assisted solution under the same conditions, enabling meaningful comparison between human and AI performance. In particular, the novelty of this work lies in extending α-angle measurement to dynamic video-based ultrasound and validating its accuracy against a phantom with a known reference angle.

## 2. Materials and Methods

### 2.1. Study 1

This study investigated the diagnostic accuracy and efficiency of Graf method ultrasound assessments across different levels of clinical experience using a standardized phantom model. Participants were divided into three groups: the trained group (T group), comprising seven individuals who had completed a specialized infant hip ultrasound seminar; the resident group (R group), consisting of seven orthopedic residents without formal ultrasound seminar training; and the student group (S group), composed of sixteen medical students without prior specialized ultrasound education.

**Figure 1:**
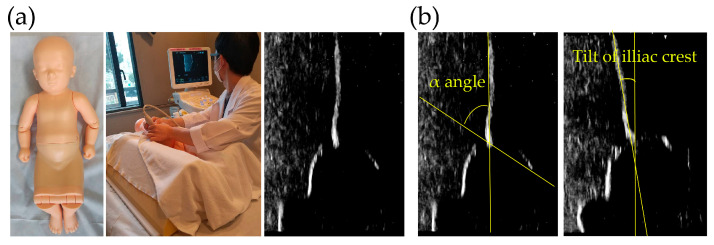
(**a**) Standardized infant hip phantom model (Kyoto Kagaku Co., Ltd., Kyoto, Japan) used for ultrasound examination and demonstration of the scanning procedure according to the Graf method. (**b**) Representative ultrasound images of the phantom hip. The α-angle is defined as the angle between the baseline along the iliac outer margin and a line extending to the bony acetabular rim. The tilt of the iliac crest relative to the vertical axis was also measured to evaluate probe positioning and image acquisition quality.

All ultrasound examinations were performed using the same ultrasound device (Hitachi-Aloka, Mitaka, Japan) and a pediatric hip phantom model (Kyoto Kagaku Co., Ltd., Japan) simulating the anatomical structure of an infant hip (Figure 1). Participants conducted the examinations according to the Graf method, with the phantom positioned in the lateral decubitus position. Each participant was instructed to align the iliac outer margin vertically, identify key anatomical landmarks, and acquire an appropriate image for subsequent α-angle measurement. A total of four independent measurements were obtained per participant.

The evaluation focused on three key parameters: the time required to obtain a diagnostic-quality image, the inclination of the iliac outer margin relative to the vertical axis, and the measured α-angle. Examination time was recorded for each trial, and the iliac inclination angle was assessed to verify correct probe positioning. α-angles were measured from the acquired images according to the standard Graf technique [12].

This study was a phantom-based experiment designed to evaluate the effect of clinical experience on operator variability. The sample size was intentionally limited to representative participants from three experience levels within our institution (trained clinicians, residents, and medical students), which was considered sufficient to demonstrate experience-related trends rather than clinical outcomes. Participants were randomly selected from available rotating clinicians and medical students during the study period. No exclusion criteria were applied, and each participant performed five independent examinations per hip joint to allow intra-operator analysis.

### 2.2. Study 2

To address the operator-dependent variability identified in Study 1, we evaluated the performance of an automated α-angle measurement system using static ultrasound images acquired from the same standardized hip phantom. A different group of orthopedic residents, not involved in Study 1, performed four independent measurements each, following the Graf method under standardized conditions. Four static ultrasound images per participant were collected for analysis.

Manual measurements of the α-angle and iliac outer margin inclination were performed by the same physicians who acquired the ultrasound images, strictly following Graf’s method. The baseline was defined along the iliac outer margin, and a second line was extended to the bony acetabular rim. The α-angle was then calculated using the built-in angle measurement tool of the ultrasound system. Each participant repeated the measurements independently, and duplicate trials per hip joint were used to assess intra-operator consistency.

#### 2.2.1. Static-Image-Based Diagnostic Algorithm for DDH

**Figure 2:**
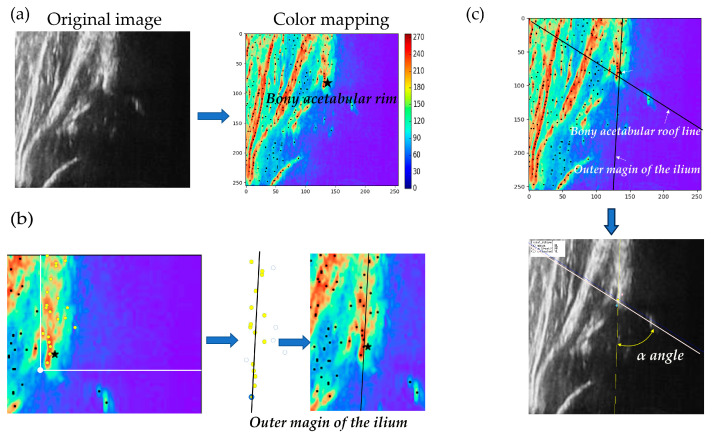
Schematic illustration of the AI-assisted α-angle measurement system. (**a**) Original ultrasound image of the hip joint and corresponding color-mapped image highlighting structural features, with the bony acetabular rim indicated. (**b**) Automatic extraction of anatomical landmarks: detection of the outer margin of the ilium and localization of the bony acetabular rim. (**c**) Determination of the baseline along the iliac outer margin and the bony acetabular roof line, followed by automated calculation of the α-angle on the reconstructed ultrasound image.

Subsequently, the same static ultrasound images were analyzed using an automated algorithm developed in accordance with a previously published method [13]. The images were first preprocessed to enhance quality and suppress noise, followed by automatic detection of key anatomical landmarks, including the bony acetabular rim using a convolutional neural network [14] (Figure 2a). After landmark detection, the system segmented relevant bone and cartilage regions and applied line fitting based on the Hough transform to construct a baseline along the iliac outer margin (Figure 2b). The α-angle was then calculated as the angle between this baseline and a line extending to the tip of the labrum (Figure 2c).

All automated measurements were generated without human intervention. For validation, manual measurements were obtained independently by the same physicians who acquired the ultrasound images, strictly following Graf’s method. Both manual and automated measurements were performed in a blinded manner, and agreement and consistency between the two approaches were subsequently evaluated.

#### 2.2.2. Dynamic-Image-Based Diagnostic Algorithm for DDH

To extend the capabilities of static image analysis to real-time ultrasound video sequences, we developed a novel diagnostic system capable of automatically extracting diagnostic-quality frames and calculating key angular measurements during continuous scanning. This system was built upon an enhanced image-processing pipeline based on our previous work [13], with critical modifications for real-time application.

**Figure 3:**
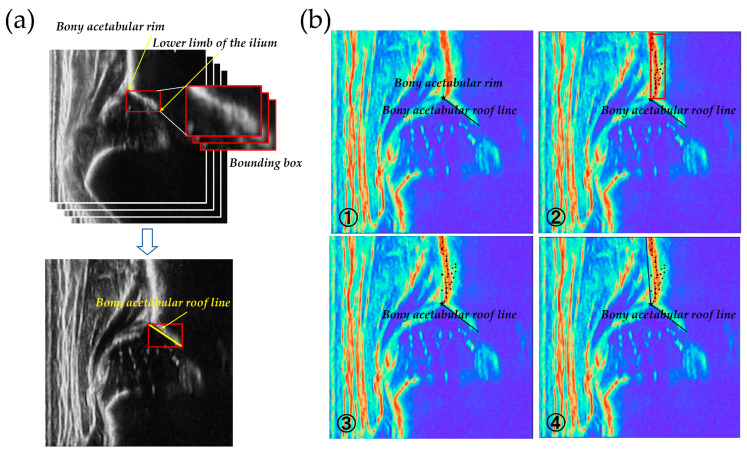
AI-assisted α-angle measurement process. (**a**) A deep learning model based on YOLO v3 was employed to perform bounding box inference on each frame, simultaneously detecting key anatomical features including the bony acetabular rim (osseous acetabular beak) and the lower limb of the ilium. (**b**) Subsequent image processing steps: (**1**) extraction of local maxima from grayscale ultrasound images to identify high-intensity bone surfaces; (**2**) screening of local maxima through intensity-based filtering to remove minor peaks and improve landmark reliability; (**3**) linear approximation using screened local maxima to construct the iliac outer wall line; and (**4**) establishment of a baseline parallel to the iliac outer margin passing through the detected acetabular rim.

First, a deep learning model based on YOLO v3 was employed to perform bounding box inference on each frame [15]. The model simultaneously detected key anatomical features, specifically the bony acetabular rim (osseous acetabular beak) and the lower limb of the ilium. By using bounding box predictions instead of traditional point detection methods, the computational burden was significantly reduced, enabling near real-time analysis (Figure 3a). Subsequent processing steps included: (1) extraction of local maxima from grayscale ultrasound images to identify high-intensity bone surfaces; (2) application of intensity-based filtering to eliminate minor peaks and improve landmark reliability; (3) linear approximation using screened local maxima to construct the iliac outer wall line; and (4) establishment of a baseline parallel to the iliac outer margin that passes through the detected acetabular rim (Figure 3b).

The α-angle was calculated in each extracted diagnostic-quality frame by measuring the angle between the constructed baseline and the identified acetabular rim line. This bounding box–based approach, combined with optimized peak screening, reduced processing time by approximately 100 to 200 milliseconds per frame compared to previous methods. These enhancements allowed for near real-time guidance during ultrasound examinations, assisting operators in identifying appropriate frames for diagnosis and enabling continuous automated assessment of hip morphology. All calculations were performed automatically without human intervention, and the results were compared with manual measurements under blinded conditions to ensure consistency.

### 2.3. Statistical Analysis

Statistical analyses were performed using JMP Pro version 17.0 (SAS Institute, Cary, NC, USA). For Study 1, group differences were assessed by one-way ANOVA. In addition to *p*-values, the observed effect size (η^2^ and Cohen’s f) and post hoc statistical power were calculated to evaluate the adequacy of the sample size. For reliability analyses, intra- and inter-operator consistency was assessed using intraclass correlation coefficients (ICCs), and 95% confidence intervals were reported together with point estimates. For Study 2, agreement with the phantom reference (70°) was evaluated by comparing mean α-angle values and the variability of measurements across methods, with bias and variability reported as the most relevant metrics. A *p*-value < 0.05 was considered statistically significant.

## 3. Results

### 3.1. Study 1

Manual Ultrasound Measurements Across Different Experience Levels in Study 1, 30 participants (7 trained clinicians, 7 residents, and 16 medical students) each performed six independent ultrasound measurements on a standardized phantom model with a known reference α-angle of 70°. The mean examination time was significantly shorter in the trained group compared to the resident and student groups (T group: 5.1 ± 2.3 s; R group: 11.4 ± 5.5 s; S group: 21.6 ± 15.0 s; *p* < 0.001), with a large effect size (η^2^ = 0.291; Cohen’s f = 0.64) and adequate post hoc power (0.85). The inclination of the iliac outer margin relative to the vertical axis was closest in the trained group, with greater deviations observed in the resident and student groups (*p* < 0.01). Intra-operator variability, assessed by intraclass correlation coefficients (ICCs), was highest in the trained group (ICC = 0.92 (95% CI: 0.89–0.95)), followed by the resident group (ICC = 0.85 (95% CI: 0.79–0.91)) and the student group (ICC = 0.78 (95% CI: 0.69–0.87)). Inter-operator reproducibility followed a similar trend (T group ICC = 0.89 (95% CI: 0.83–0.95); R group ICC = 0.81 (95% CI: 0.74–0.87); S group ICC = 0.74 (95% CI: 0.69–0.79)).

### 3.2. Study 2: Comparison of Manual and Automated α-Angle Measurements

#### 3.2.1. Manual Measurements

The mean α-angle measured manually was 64.0° (SD = 4.7°), substantially underestimating the reference value of 70°. Bland–Altman analysis indicated a mean bias of −6.0°, with 95% limits of agreement ranging from −15.2° to +3.2°, confirming systematic underestimation by manual measurement.

#### 3.2.2. Static-Image-Based Automated Measurements

The static-image-based diagnostic algorithm yielded a mean α-angle of 69.3° (SD = 10.4°). Although the mean value closely approximated the reference, Bland–Altman analysis against the phantom reference (70°) showed a small mean bias of −0.7°, but with wide 95% limits of agreement (−21.1° to +19.7°), indicating considerable variability despite acceptable mean accuracy. Error distribution plots confirmed the wide dispersion of static-image measurements around the reference, reflecting limited reproducibility.

#### 3.2.3. Dynamic-Image-Based Automated Measurements

**Figure 4:**
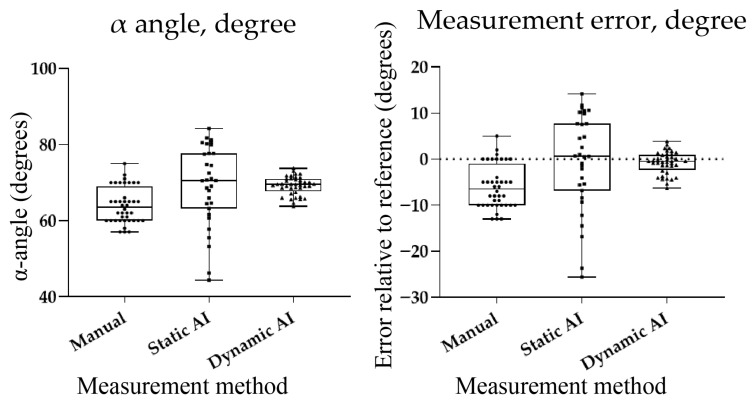
Comparison of manual, static AI, and dynamic AI measurements of the α-angle using a phantom model. (**Left**): Distribution of α-angle measurements. The dashed horizontal line indicates the true reference value (70°). Manual measurements underestimated the reference, static AI approximated the reference but with large variability, and dynamic AI clustered tightly around the true value. (**Right**): Distribution of measurement errors relative to the reference value (70°). Dynamic AI demonstrated the lowest median error and smallest variability, highlighting its superior accuracy and reproducibility compared with manual and static AI.

The dynamic-image-based diagnostic system analyzed continuous ultrasound video sequences. The mean α-angle measured from automatically extracted diagnostic-quality frames was 69.2° (SD = 2.4°), demonstrating both high accuracy and low variability. Bland–Altman analysis against the phantom reference (70°) showed a mean bias of −0.8°, with narrow 95% limits of agreement (−5.5° to +3.9°), indicating excellent agreement and reproducibility. Compared to static-image analysis, the dynamic system achieved greater consistency and precision. The diagnostic success rate was 100% (40/40 frames correctly assessed), with a mean absolute error of 0.8° relative to the reference value. The processing time per frame was reduced by approximately 100–200 milliseconds, enabling near real-time assessment. Error distribution plots confirmed that dynamic AI measurements were tightly clustered around the true value with minimal variance, whereas manual measurements systematically underestimated and static-AI assessments exhibited wide variability (Figure 4).

## 4. Discussion

Early and accurate diagnosis of DDH is critical for preventing long-term sequelae and reducing the need for invasive interventions [16]. Although ultrasound examination using the Graf method has become the standard for infant hip screening 16, its reliability remains significantly influenced by examiner technique. Recent systematic reviews and studies have emphasized persistent intra- and inter-operator variability in α- and β-angle measurements, even among trained practitioners [17,18,19]. Minor deviations in probe angulation, pressure, and landmark identification can significantly impact diagnostic outcomes, highlighting the inherent difficulty of achieving fully reproducible manual assessments.

Our first study confirmed that operator variability persists across different levels of clinical experience. Trained clinicians demonstrated significantly shorter examination times, better iliac margin alignment, and more consistent α-angle measurements compared to residents and medical students. These findings align with previous reports showing that even novice operators can achieve high reproducibility after brief, structured training programs, typically consisting of 1.5–2 h of instruction and supervised practice [3,7,20]. Intraclass correlation coefficients exceeding 0.9 have been reported after such interventions [3,7]. However, our results also suggest that a major source of measurement variability may lie not only in the angle measurement itself, but in the ability to acquire an appropriate standard plane for evaluation. Since α-angle accuracy is inherently dependent on the quality of the acquired image, especially the alignment of the iliac margin and identification of key landmarks, acquiring the correct standard plane appears to be a particularly critical and experience-sensitive skill. Supporting operators in obtaining such images may therefore be essential to improving reproducibility in DDH screening.

To address these limitations, several strategies have been proposed. Immediate feedback during scanning, continuous quality assurance programs, and refresher training are essential to sustain diagnostic accuracy and minimize technical drift over time. Common error sources, such as misalignment of the iliac margin and incorrect landmark identification, continue to affect measurement reliability, particularly among less experienced operators. Adjunctive technologies, including computer-aided diagnosis (CAD) systems and three-dimensional (3D) ultrasound, have shown promise in further reducing operator-dependent variability [4,21]. These tools can provide real-time feedback, enhance standardization, and support novice examiners in acquiring diagnostic-quality images.

Building on these observations, we evaluated an AI-assisted system for automated α-angle measurement using static ultrasound images and dynamic video sequences. The system showed high accuracy: static-image results closely matched the reference, and dynamic analysis achieved even greater consistency with narrower Bland–Altman limits of agreement. These findings support the utility of AI in DDH screening, where prior studies have reported classification accuracies of 90–98% and mean absolute errors of 1.7–2.5°. Compared to earlier methods such as that by Chen et al. [22], our system focuses specifically on α-angle measurement—central to early DDH diagnosis—and enables real-time processing by combining bounding box inference with local peak detection. Phantom-based validation with a known reference angle allowed for the objective assessment of measurement error, independent of clinical variability. A principal strength of this study is the novelty of applying a dynamic, video-based AI system for α-angle measurement, moving beyond previously reported static-image approaches. This real-time capability is particularly relevant to clinical practice, as continuous frame analysis can support consistent identification of diagnostic-quality planes. Moreover, validation against a phantom with a known reference angle provides a robust and objective benchmark, reinforcing the reliability of both human and AI performance. Although clinical validation is still needed, these results suggest that AI tools may improve reproducibility and standardization in ultrasound-based DDH screening.

Several limitations of this study should be acknowledged. First, the validation of the AI-assisted system was conducted using a phantom model rather than clinical ultrasound images. Although the use of a phantom with a known reference α-angle enabled objective quantification of measurement error, the performance of the system in real-world clinical settings remains to be evaluated. Clinical images typically present greater variability due to patient movement, anatomical diversity, and differences in operator technique, which could affect the system’s accuracy and robustness. Second, the number of participants involved in the manual ultrasound assessments was relatively limited. While significant differences were observed between groups with different levels of experience, larger sample sizes across diverse institutions would be necessary to generalize the findings regarding operator-dependent variability. Third, the AI algorithm focused exclusively on α-angle measurement without incorporating β-angle evaluation. Although α-angle is the primary determinant for early DDH classification, comprehensive assessment following Graf’s original methodology also considers β-angle measurements, which may contribute additional diagnostic information in certain cases. Finally, this study did not directly assess the system’s usability or integration within real-time clinical workflows. Future work should include prospective clinical trials to evaluate not only diagnostic accuracy but also the practical impact on workflow efficiency, examiner confidence, and training effectiveness when AI-assisted tools are deployed in routine DDH screening programs. Future work should therefore include prospective clinical trials to evaluate not only diagnostic accuracy but also the practical impact on workflow efficiency, examiner confidence, and training effectiveness when AI-assisted tools are deployed in routine DDH screening programs, as well as investigations of system performance under real-world conditions of patient motion, anatomical variability, and variable image quality. Moreover, extending the algorithm to incorporate β-angle analysis and optimizing it for seamless integration into daily practice will be important next steps to maximize clinical applicability.

## 5. Conclusions

This study confirmed that substantial operator variability persists in infant hip ultrasound despite structured training, with trained clinicians achieving shorter examination times and more reproducible measurements than residents and medical students. Using a phantom model with a known reference α-angle, we demonstrated that manual measurements systematically underestimated the true value, static AI achieved closer estimates with higher variability, and dynamic AI provided the highest accuracy and consistency with near real-time performance. Compared to previous frameworks, our system therefore offers improved reproducibility and real-time diagnostic support, focusing on the most clinically critical parameter. In practical terms, these findings suggest that integrating AI-assisted α-angle measurement into routine DDH screening could reduce operator dependence, improve diagnostic reproducibility, and support the training of less experienced examiners. Future clinical validation will be essential to establish its broader applicability for standardized DDH screening.

## 6. Patents

A Japanese patent application related to the methodology described in this manuscript has been filed by the authors.

## Data Availability

The data presented in this study are available on request from the corresponding author.

## Abbreviations

The following abbreviations are used in this manuscript:

DDH	Developmental Dysplasia of the Hip
AI	Artificial Intelligence
ICC	Intraclass Correlation Coefficient
YOLO	You Only Look Once (deep learning model)
3D	Three-Dimensional
CAD	Computer-Aided Diagnosis
